# Captive wildlife management survey in Vietnam, 2015–2021

**DOI:** 10.1016/j.onehlt.2023.100543

**Published:** 2023-04-17

**Authors:** Nhu Van Thu, Scott Newman, Pawin Padungtod

**Affiliations:** a Emergency Center for Transboundary Animal Diseases, Food and Agricultural Organization of the United Nations, Country Office for Vietnam, Hanoi, Viet Nam; b Food and Agricultural Organization of the United Nations, Regional Office for Asia and the Pacific, Bangkok, Thailand

**Keywords:** Endangered species, Vietnam, Breeding, Captive wildlife farming, Management software, one health

## Abstract

In Vietnam, breeding and raising a wide range of wildlife species in captive wildlife facilities (CWFs) are common practices but little information on the captive wildlife population is available. We conducted surveys and developed software to create a captive wildlife facilities management (CWFM) system. This database provides up-to-date information on the distribution of CWFs, the number of species, and individuals according to the level of protection outlined by the government and the Convention on International Trade in Endangered Species of Wild Fauna and Flora (CITES) categories. CWFs were located in all provinces and regions, but differed in distribution, number of species and individual animals. The Mekong River Delta region recorded the highest number of CWFs (35.3%) and the highest number of animals (43.1%). In 2021, 95 species belong to the highest level of protection group were being raised at 1824 CWFs; 137 species in 4554 CWFs in CITES appendix II, appendix III, government list IIB; and 139 species in 1499 CWFs belong to the common wildlife. The overall number of CWFs in 50 provinces decreased by a negative compound annual growth rate of −7.2%. However, it is crucial to continue to monitor the changing dynamics to assess the risks of disease transmission from zoonoses originating from wildlife. We recommend periodic compulsory reporting of CWF activities using the CWFM system.

## 1 Introduction and purpose

In Vietnam, breeding, raising and commercial trading of a wide range of captive wildlife species, including insects, amphibians, reptiles, birds, and mammals, are common practices [1,2]. Commercial trading refers to the practice of raising of a wildlife species that is capable of breeding in captivity with the intention of harvesting the animal or an animal product for commercial profit [3]. Captive wildlife husbandry has a greater risk than conventional livestock farming due to limited knowledge of the farmer about wildlife nutritional requirements, their behavior, biological characteristics, diseases, and environmental adaptability [4,5]. It requires large financial investment and faces an unstable market, with supply often outgrowing demand [5,6]. Additionally, there is a lack of adequate state orientation, management, and operating strategy [4]. However, breeding and raising some species can be profitable and attracts many partakers [7]. Year by year, the number of captive wildlife facilities (CWFs), involving animals such as snakes, ostriches, crocodiles, porcupine, and wild boars, fluctuates [5,[7], [8], [9], [10]].

The Government of Vietnam (GoV) has issued a number of laws and regulations to improve wildlife protection and is a signatory to the Convention on International Trade in Endangered Species of Wild Fauna and Flora (CITES) [11]. The wildlife classification and levels of protection were updated in 2021 in decree 84/ND-CP/2021 [12] to cover all terrestrial species included in the previous decrees (06–2019 [13], 64/2019 [14] and the CITES appendices [16]. These classifications and groupings do not completely align because the decree 84 does not include all species of exotic animals, and the CITES appendices do not include all species of local wildlife. To harmonize those lists, the highest prioritized protection scheme, Level 1, covers endangered, rare, and precious species with a high risk of extinction (CITES appendix I [16], Government list IB [12] and decree 64/2019) [14]. Level 2 covers species not yet at a high risk of extinction, but those that without protective measures, would be at risk (CITES appendix II [16], Government list IIB [12]. Level 3 covers common and other wildlife that are not covered under CITES. The remaining animals belong to livestock and other wild animals not under the management of the Vietnam Administration of Forestry (VNForest) [12].

The GoV has requested provincial sub-departments of forest protection (sDFPs) to regularly report information on CWFs (GoV, 2021). However, collecting data from the CWFs is difficult due to the financial and human resources needed to monitor the large number of scattered facilities, some with few individual animals. In 2014, the VNForest, in collaboration with the Food and Agricultural Organization of the United Nations (FAO) Vietnam office, conducted a pilot survey on CWFs using a newly developed survey tool in 12 provinces [18]. The survey identified 4099 operating CWFs and captured information on 1,554,511 animals. There were 1,218,547 animals belonging to 182 wildlife species, plus 335,964 cattle, poultry and other domestic species raised in these CWFs.

To improve the monitoring and evaluation of CWFs, the inventory of CWFs needed to be updated and expanded. With more efficient data management, the scope and range of wildlife breeding and raising in Vietnam could be identified. Under the One Health Partnership (OHP) Framework, a Technical Working Group (TWG) on Wildlife and Pandemic Prevention has been established to reduce the likelihood of future pandemic risk. The focus is on facilitating better management of CWFs trade and consumption, reducing fraudulent practices, and minimizing public health impact from zoonoses originating from wildlife.

The CITES Management Authority of Vietnam (CITES MA), under the VNForest, in collaboration with FAO Vietnam office, conducted surveys and developed software to create a captive wildlife facilities management (CWFM) system that would provide up-to-date information on the distribution of CWFs, herd structure, reproduction abilities, the number of wildlife species and individual animals in the CWFs for planning, monitoring and communication campaign, details of these wildlife species according to the GoV and CITES categories, and the fluctuations of CWFs.

## 2 Methods

We revised the original survey tools developed in 2015 that were GoV's national legal template for annual reporting. In 2015 we collected data from 12 provinces and cities. In 2017, we collected data from 11 new provinces and updated the information from the original 12 provinces. Thirty-five other provinces reported their data to CITES-MA using similar reporting template.

In 2020, we revised the survey tool and collected data in 61 provinces. We developed software to manage the CWFM database. The database had four main components: 1) data entry of information on owners of CWFs; 2) data entry of information about wildlife species at the CWFs; 3) generation of detailed and summary reports; and 4) system and user management functions. Identification of captive wildlife species was based on record books and profiles declared by farmers and local forestry agencies. Each species was encoded, using its scientific name, and checked with synonyms to ensure unique names and uniform reporting among all the provinces.

The survey tool and database management software were piloted by the sDFPs in three initial provinces of Nghe An, Dong Nai and Bac Lieu in 2020. In February 2021, we expanded the piloting of the survey tool and database management software to five other provinces of Long An, Dong Thap, Tay Ninh, Binh Thuan, and Binh Phuoc. Training for data collection and use of the software was then organized for all 63 provincial sDFPs and district station staff from May–October 2021. In 2021 we collected data in 54 provinces.

All CWFs under the management of VNForest were required to collect and enter data into the system. With the support from CITES-MA and FAO, the data of each year from 2017, 2020 and 2021 was collected up to 31 December and entered into the CWFM database. We extracted the data and transferred it to MS-Excel and MS-Access. Summary statistics were calculated using MS-Excel. The distribution maps were developed with ARC-Map software with WGS-84 UTM coordinates. Animals were categorized based on the GoV and CITES level of protection and wildlife animal groupings [[12], [13], [14], [15],^17^]. To evaluate the fluctuation of CWFs over the years, a compound annual growth rate (CAGR) was used for assessing three criteria: the number of facilities (CAGRf), number of individuals (CARGi), and number of provinces (CARGp). The formula for the CAGR calculation was: (V_b/V_f)1 / t-1, where CAGR = compound annual growth rate; V_b = beginning value; V_f = final value; and t = time in years.

## 3 Results

**Fig. 1:**
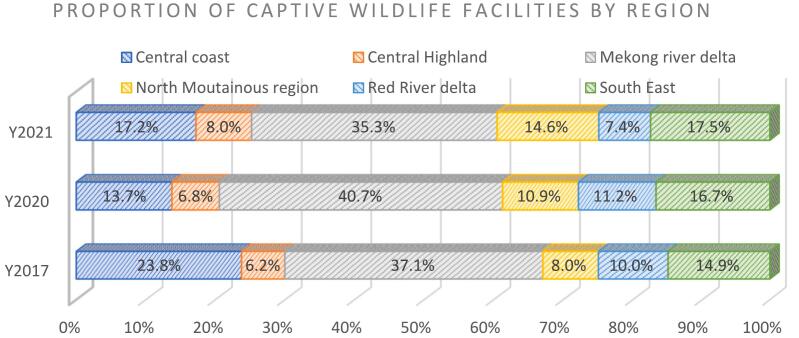
Proportion of captive wildlife facilities by regions, Vietnam: 2017, 2020 and 2021.

CWFs were located in all provinces and regions, but they differed in distribution, number of species and individual animals. The Mekong River Delta (MRD) region recorded the highest number of CWFs (35.3%), followed by the Southeast (SE) at 17.5% and the Central Coast (CC) 17.2%. The Central Highland (CH) and Red River Delta (RRD) were the two regions with the fewest number of CWFs. The four provinces in 2021 with >300 CWFs were Dong Nai (668), Dak Lak (449), Nghe An (355) and Ca Mau (387) (Fig. 1).

**Fig. 2:**
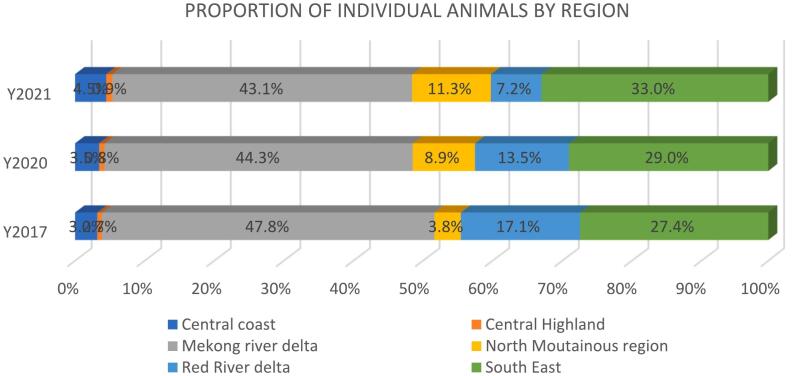
Proportion of individual animals by regions, Vietnam: 2017, 2020 and 2021.

In 2020 the MRD recorded the highest number of individual animals (43.1%), followed by the SE region (33.0%), and the Northern Mountainous (NM) region (11.3%). The CH and RRD were the two regions with the fewest number of individual animals. There were three provinces where >80 species were raised in CWFs, including Ho Chi Minh City (104), Khanh Hoa (64) and Binh Duong (41). The four provinces with the most individual animals were Dong Nai (335,197), Dong Thap (261,688) and Bạc Liêu (206,016) (Fig. 2).

In the analysis of data from 2017, 2020 and 2021, the distribution of CWFs and individual wildlife varied between regions. The MRD region recorded the highest proportion of CWFs and individual animals in all three years. It was followed by the SE region and the NM region (Table 1).

**Table 1:** Distribution of captive wildlife facilities and individual wildlife by regions, Vietnam: 2017, 2020, and 2021.

*Region and year*	*Number of Facilities*	*Number of Animals*	*Proportion by facilities*	*Proportion by animals*
*2017*				
*Central Coast (CC)*	*2875*	*93,635*	*23.8%*	*3.2%*
*Central Highland (CH)*	*751*	*19,392*	*6.2%*	*0.7%*
*Mekong River Delta (MRD)*	*4489*	*1,399,662*	*37.1%*	*47.8%*
*North Mountainous (NM)*	*964*	*110,647*	*8.0%*	*3.8%*
*Red River Delta (RRD)*	*1213*	*501,407*	*10.0%*	*17.1%*
*South East (SE)*	*1806*	*803,756*	*14.9%*	*27.4%*
*Total = 58 provinces*	*12,098*	*2,928,499*		
*2020*				
*Central Coast (CC)*	*1090*	*84,294*	*13.7%*	*3.5%*
*Central Highland (CH)*	*542*	*18,847*	*6.8%*	*0.8%*
*Mekong River Delta (MRD)*	*3229*	*1,078,849*	*40.7%*	*44.3%*
*North Mountainous (NM)*	*864*	*217,452*	*10.9%*	*8.9%*
*Red River Delta (RRD)*	*885*	*328,534*	*11.2%*	*13.5%*
*South East (SE)*	*1322*	*707,169*	*16.7%*	*29.0%*
*Total = 61 provinces*	*7932*	*2,435,145*		

*2021*				
*Central Coast (CC)*	*1161*	*83,839*	*17.2%*	*4.5%*
*Central Highland (CH)*	*539*	*16,319*	*8.0%*	*0.9%*
*Mekong River Delta (MRD)*	*2379*	*805,336*	*35.3%*	*43.1%*
*North Mountainous (NM)*	*986*	*211,652*	*14.6%*	*11.3%*
*Red River Delta (RRD)*	*502*	*135,522*	*7.4%*	*7.2%*
*South East (SE)*	*1177*	*616,767*	*17.5%*	*33.0%*
*Total = 54 provinces*	*6744*	*1,869,435*		

The analysis of data from 2015, 2017, 2020, and 2021 identified 454 species of wild animals. The surveys recorded 308 species in 59 provinces in 2017, 388 species in 61 provinces in 2020, and 371 species in 54 provinces in 2021. Of these, 148 species were recorded in all years; 98 species were recorded in any three years; 147 species were recorded in any two years, and 61 species were recorded only once.

In 2021, the system recorded 385 animal species, of which 371 species were considered as wild animals under the management of VNForest. These wild species belonged to five classes (Amphibians, Arachnida, Aves, Mammalia and Reptilia), 36 orders and 102 families. There were 128 species of mammals with 156,530 individuals; 151 species of birds with 45,065 individuals; and 81 species of reptiles with 1,662,959 individuals. (Table 2).

**Table 2:** Classification and population of wildlife in Vietnam, 2021.

*Class*	*#order*		*#family*		*#species*		*#herds*		*#population*		*% in facilities*	*% in population*
	*T*⁎	*C*†	*T*	*C*	*T*	*C*	*T*	*C*	*T*	*C*		
*Amphibians*	*2*	*1*	*8*	*3*	*8*	*3*	*10*	*5*	*4861*	*4849*	*0.11%*	*0.26%*
*Arachnida*	*2*	*2*	*2*	*2*	*3*	*2*	*3*	*2*	*20*	*6*	*0.03%*	*0.00%*
*Aves*	*18*	*16*	*34*	*21*	*151*	*76*	*673*	*376*	*45,065*	*40,282*	*7.34%*	*2.41%*
*Mammalia*	*11*	*10*	*36*	*25*	*128*	*58*	*4857*	*4433*	*156,530*	*149,776*	*52.97%*	*8.37%*
*Reptilia*	*3*	*3*	*22*	*18*	*81*	*58*	*3626*	*3422*	*1,662,959*	*1,635,847*	*39.55%*	*88.96%*
*Total*	*36*	*32*	*102*	*69*	*371*	*197*	*9169*	*8238*	*1,869,435*	*1,830,760*		

⁎ T = Total captive wildlife facilities.

† C = Commercial facilities.

Reptiles, including crocodiles, pythons, snakes, and turtles, were the most numerous species. In total there were 1,662,959 (88.9%) individuals in 3626 (39.5%) CWFs. The region with the most reptiles was MRD, with 780,612 (41.8%) individuals in 1699 (18.5%) CWFs.

Mammals were raised in 52.9% of CWFs that were located in CC (13%), SE (10.5%) and MRD (10.3%). However, the total number of mammals accounted for only 156,530 (8.4%) of all individuals. The largest number of mammals were in the SE (3.1%), MRD (2.1%), and CC (1.4%).

All animal classes were identified in CWFs in all provinces. Among the CWFs recorded from 2017 to 2021, 77.8%–88.3% raised only one species of wild animal, 10.2%–11% raised 2–4 species, and 1.2%–1.5% raised five species or more. This data excludes information about other livestock, pets, and non-monitored wildlife. In 2021, there were 197 species (53.1%) with 1,830,760 (97.9%) individuals being raised at 6689 CWFs. Aves accounted for 76 species (40,282 individuals); mammals for 58 species (149,776 individuals) and reptiles for 58 species (1,635,847 individuals).

In 2021, there were 76 bird species belonging to 16 orders, 21 families and 392 flocks.

for a total of 40,580 individuals raised in 288 CWFs. The Phasianidae family of the order.

Galliformes dominated with a presence of 233 flocks (65.9%% 190/288) with 22,600 individuals raised in 190 CWFs. The Green Peacock *(P. cristatus)* were raised at 108 CWFs (37.5% 108/288) with 2514 individuals, followed by the Ring-necked pheasant *(P. colchicus)* raised at 59 CWFs (20.4%%, 59/288) with 16,441 individuals.

For the mammal class, there were 58 species being raised in 4433 CWFs with 149,776 individuals. The carnivora, artiodactyla and rodentia dominated the number of CWFs. Typical species included the Asian palm civet (1570 CWFs, 23,344 individuals), sambar deer (992 CWFs, 4328 individuals), porcupines (571 CWFs, 16,103 individuals), bamboo rat (399 CWFs, 29,750 individuals) and wild boar (217 CWFs, 7499 individuals). Primates were kept in a limited number of CWFs (52), but some species were kept in large commercial farms for research purposes. These included the Long-tailed macaque (*M. fascicularis*) in 24 CWFs with 44,123 individuals and the Rhesus macaque (*M. mulatta*) in 7 CWFs with 1126 individuals.

There were 95 endangered, rare, and precious species of wild animals under Level 1 protection, kept in 1524 CWFs, with 943,351 individuals. There were 137 wildlife species not yet at a high risk of extinction, but that needed protection under Level 2 protection, kept in 4554 CWFs, with 697,274 individuals. There were 139 common wildlife species under Level 3 protection, kept in 1499 CWFs, with 228,810 individuals. According to the CITES Appendix, in 2021 there were 210 species (56.6% of wildlife) kept in 4579 CWFs with a total of 1,634,958 individuals (87.5% of wildlife) included in three appendices. (Table 3).

**Table 3:** Vietnamese Government and CITES levels of protection for wildlife species, 2021.

*Level of protection*	*Protection* *Regulation*	*#* *Species*	*#* *Facilities*	*#* *Herds*	*#* *Individuals*
*Government decree 84*–*2021*		*371*	*6744*	*9169*	*1,869,435*
*Level 1*⁎	*Appendix IB*	*68*	*1503*	*1743*	*942,808*
	*Other-CITES-I*	*27*	*21*	*78*	*543*
	*Sum of level 1*	*95*	*1524*	*1821*	*943,351*
*Level 2*†	*Appendix IIB*	*73*	*3761*	*4449*	*460,787*
	*Other CITES-II*	*57*	*662*	*783*	*233,340*
	*Other CITES-III*	*7*	*131*	*138*	*3147*
	*Sum of level 2*	*137*	*4554*	*5370*	*697,274*
*Level 3*‡	*Common wildlife*	*139*	*1499*	*1978*	*228,810*
*Government Decree 64*–*2020*					
*Level 1*	*List of endangered, rare, and precious animals*	*59*	*205*	*395*	*3712*
*CITES Appendix*		*210*	*4579*	*6104*	*1,634,958*
*Level 1*	*Appendix I*	*66*	*1451*	*1660*	*941,314*
*Level 2*	*Appendix II*	*130*	*1592*	*2466*	*661,539*
	*Appendix III*	*14*	*1814*	*1978*	*32,105*
*Level 3*	*Other wildlife (not listed)*	*161*	*2977*	*3065*	*234,477*
*Level 4*$*: Wildlife/animal species* *not managed*		*14*	*561*	*591*	*80,831*
*Total*		*385*	*7184*	*9760*	*1,950,266*

⁎ Level 1 = endangered, precious, and rare species prioritized for protection (Appendix IB of decree 84–2021 or CITES Appendix I or decree 64–2019).

† Level 2 = wildlife species that needed protection in Appendix IIB in the decree 84/2021 or Appendix II, III of CITES.

‡ Level 3 = common wildlife and other wildlife according to decree 84/2021, not included in the CITES appendices.

$ Level 4 = livestock or species not managed by the forestry sector (decree 13/2020 or decision 4737–2021.

The analysis of data from all four years found that 285 species were indigenous to Vietnam and 165 were exotic imported species. According to the origin and natural habitat of the 371 wildlife species being bred or raised in Vietnam in 2021, approximately 135 species (36.4%), with a total of 7214 individuals, were exotic; either imported or with no history of natural distribution in Vietnam. This exotic species group was mainly raised at zoos, experimental breeding facilities, or kept as pets. The other 232 species (62.5%) were found to be naturally distributed in Vietnam. Of these, 70 species belong to Level 1, 82 species to Level 2, and 30 species to Level 3 of protection according to CITES. There are approximately 1,862,154 (99.6%) individuals classified as native animals according to Decree 84 (Table 4).

**Table 4:** Distribution of native and exotic wildlife species under CITES and Decree 84 protection levels in Vietnam, 2021.

*Protection group*	*Sources/ known distribution*	*# Species*	*# Herds*	*# Individuals*
*Level 1*⁎	*Native*	*70*	*1745*	*942,821*
	*Exotic*	*25*	*76*	*530*
*Level 2*†	*Native*	*82*	*5108*	*693,380*
	*Exotic*	*55*	*262*	*3894*
*Level 3*‡	*Native*	*80*	*1860*	*225,953*
	*Unknown*	*4*	*4*	*67*
	*Exotic*	*55*	*114*	*2790*
*Total local native wildlife*		*232*	*8713*	*1,862,154*
*Total exotic/imported wildlife*		*135*	*452*	*7214*
*Total wildlife*		*371*	*9169*	*1,869,435*

⁎ Level 1 = endangered, precious, and rare species prioritized for protection (Appendix IB of decree 84–2021 or CITES Appendix I or decree 64–2019).

† Level 2 = wildlife species that needed protection in Appendix IIB in the decree 84/2021 or Appendix II, III of CITES.

‡ Level 3 = common wildlife and other wildlife according to decree 84/2021, not included in the CITES appendices.

Data updated from 54 provinces with 6744 CWFs in 2021 showed that in the Level 1 protection group, there were 95 species being raised at 1507 facilities. The most numerous species were: freshwater crocodile *(C. siamensis)*, accounting for 86.3% (1301) CWFs in 31 provinces; black bear *(U. thibetanus),* accounting for 7.0% (105) of CWFs in 25 provinces; and green peafowl (*P. muticus*), accounting for 4.2% (63) of CWFs in 24 provinces. For the 137 species in Level 2 protection group, the most commonly raised species in CWFs were: Asian palm civet (*P. hermaphroditus*), accounting for 37.4%% (1580) of CWFs in 53 provinces; the Samba deer (*C. unicolor*), accounting for 24% (1013) of CWFs in 32 provinces; and the Indian cobra (*N.naja*), accounting for 13.7% (579) of CWFs in 34 provinces. In Level 3 protection group, the most numerous species were porcupines (*H.brachyura*), accounting for 39.0% (585) of CWFs in 48 provinces (Table 5).

**Table 5:** Common species bred and raised in Vietnam and the Government and CITES protection level, 2021.

*Protection levels*	*Species*	*Total CWFs*⁎ *(371 species)*			*Commercial facilities* *(209 species)*		
		# *Pro*†	# *Faci*‡	# *Individual*	# *Pro*	# *Faci*	# *Individual*
*Level 1*$	*Crocodile (C. siamensis)*	*31*	*1301*	*937,455*	*28*	*1276*	*915,223*
	*Asian black bear* *(U. thibetanus)*	*25*	*105*	*516*	*21*	*91*	*304*
	*Green peafowl (P. muticus)*	*24*	*63*	*864*	*21*	*49*	*646*
	*Elephant (E. maximus)*	*11*	*41*	*70*	*2*	*27*	*30*
	*Monitor (V. nebulosus)*	*11*	*18*	*263*	*6*	*14*	*263*
	*Level 1 Total*	*46*	*1507*	*943,351*	*44*	*1465*	*918,583*
*Level 2*¶	*Palm civet* *(P. hermaphroditus)*	*53*	*1580*	*23,404*	*53*	*1570*	*23,344*
	*Samba deer (C. unicolor)*	*32*	*1013*	*4654*	*29*	*992*	*4328*
	*Indian cobra (N. naja)*	*34*	*579*	*219,789*	*33*	*576*	*219,773*
	*Oriental rat snake* *(P. mucosus)*	*35*	*409*	*131,999*	*34*	*407*	*131,991*
	*Level 2 Total*	*54*	*4222*	*697,274*	*54*	*4173*	*689,921*
*Level 3*#	*Porcupine (H. brachyura)*	*48*	*585*	*16,358*	*48*	*571*	*16,103*
	*Bamboo rat (R. pruinosus)*	*45*	*401*	*29,779*	*45*	*399*	*29,750*
	*Wild boar (S.scrofa)*	*36*	*222*	*7596*	*35*	*217*	*7499*
	*Chinese bamboo rat* *(R. sinensis)*	*20*	*95*	*12,166*	*20*	*95*	*12,166*
	*Level 3 Total*	*50*	*1499*	*228,810*	*50*	*1472*	*224,064*
	*Total*		*6744*	*1,869,435*		*6689*	*1,832,568*

⁎ CWFs = Captive Wildlife Facilities.

† Pro = Provinces.

‡ Faci = Facilities.

$ Level 1 = endangered, precious, and rare species prioritized for protection (Appendix IB of decree 84–2021 or CITES Appendix I or decree 64–2019).

¶ Level 2 = wildlife species that needed protection in Appendix IIB in the decree 84/2021 or Appendix II, III of CITES.

# Level 3 = common wildlife and other wildlife according to decree 84/2021, not included in the CITES appendices.

**Fig. 3:**
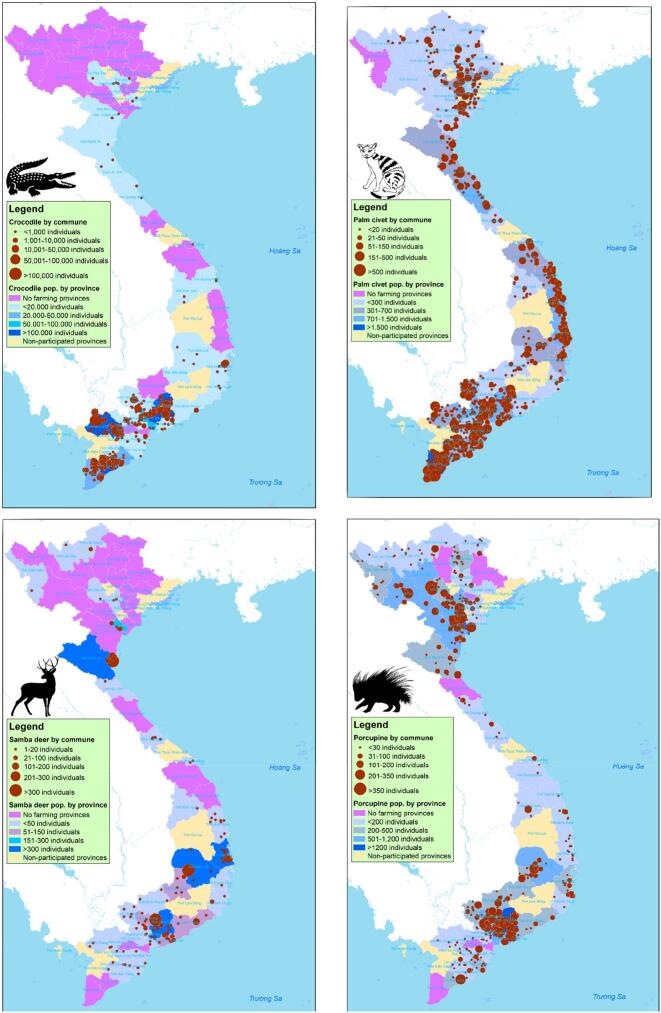
Distribution of common captive wild animals by commune and province, Vietnam, 2021.

In 2021 four of the most common species in communes and provinces were the freshwater crocodile (*C.siamensis*), the Asian palm civet (*P. hermaphroditus*), the porcupine (*H.brachyura*), and the Samba deer (*C. unicolor*). The freshwater crocodile was distributed mostly in the SE and MRD. The Asian palm civet and porcupine were raised in all provinces. The Samba deer were concentrated in Dong Nai, Dak Lak and Nghe An Province (Fig. 3).

Based on the compound annual growth rate (CAGR) from 2015 to 2021 in 12 cities and provinces, the trend of breeding and raising wild boars decreased (−35.7% in CWFs and − 34.6% in individual animals) and in porcupines (−28.3% in CWFs and − 18.5% in individual animals). However, this trend increased in Asian palm civets in 2020 (+19.6%) and 2021 (+18.1%) compared to 2017. For freshwater crocodiles, there were 520 CWFs in the region at the peak in 2017, but as of mid–2021, the number of CWFs decreased to 312 for a compound annual growth rate of facilities (CAGRf) of 12%. (Table 6).

**Table 6:** Trend of breeding and raising wildlife in 11 provinces, Vietnam: 2015, 2017, 2020, and 2021.

	*Number of CWFs*⁎				*Number of individuals*				*CARG*†	
*Species*	*2015*	*2017*	*2020*	*2021*	*2015*	*2017*	*2020*	*2021*	*Facility*	*animals*
*Malayan porcupine* *(H. brachyura)*	*1117*	*536*	*143*	*144*	*20,338*	*12,371*	*5245*	*6045*	−*28.9%*	−*18.3%*
*Oriental rat snake* *(P. mucosus)*	*494*	*222*	*156*	*124*	*84,775*	*43,933*	*70,965*	*67,521*	−*20.6%*	−*3.7%*
*Siamese crocodile* *(C. siamensis)*	*383*	*519*	*477*	*306*	*290,202*	*267,072*	*395,556*	*307,700*	−*12.4%*	*3.6%*
*Sambar deer* *(C. unicolor)*	*350*	*324*	*237*	*220*	*2080*	*1857*	*1345*	*1245*	−*7.4%*	−*8.2%*
*Wild boar* *(S. scrofa)*	*240*	*105*	*19*	*17*	*5946*	*3776*	*1191*	*410*	−*35.7%*	−*36.0%*
*Ring-necked Pheasant* *(P. colchicus)*	*198*	*77*	*22*	*25*	*14,320*	*5723*	*14,966*	*10,138*	−*29.2%*	−*5.6%*
*Indian python* *(P. molurus)*	*147*	*117*	*51*	*66*	*7669*	*13,530*	*14,087*	*15,204*	−*12.5%*	*12.1%*
*Asian palm civet* *(P. hermaphroditus)*	*130*	*151*	*264*	*421*	*2180*	*2560*	*4237*	*6433*	*21.6%*	*19.8%*
*Asian black bear* *(U. thibetanus)*	*87*	*62*	*35*	*29*	*407*	*268*	*114*	*77*	−*16.7%*	−*24.2%*
*Bengal monitor* *(V. bengalensis)*	*70*	*42*	*17*	*13*	*5171*	*1427*	*624*	*256*	−*24.5%*	−*39.4%*
*Hoary Bamboo Rat* *(R. pruinosus)*	*61*	*45*	*71*	*86*	*2237*	*1797*	*4107*	*4768*	*5.9%*	*13.4%*

⁎ CWFs = Captive wildlife facilities.

† CARG = compound annual growth rate.

Analysis from 50 provinces and cities showed that the number of CWFs gradually decreased from 8797 in 2017 to 6525 in 2021 for a CAGRf of −7.2%. The number of animals also decreased with a compound annual growth rate of individuals (CAGRi) of - 5.3%. However, some common species in >100 CWFs, or located in >20 provinces, increased. For example, the Blue peafowl (*P. cristatus*) had a compound annual growth rate of provinces (CARGp) of +24.3%. (Table 7).

**Table 7:** Trends of breeding and raising wildlife species in 50 provinces in Vietnam: 2017 and 2021.

	*Species*	*2017*			*2021*			*CAGR*⁎		
		*#* *Pro*†	*#* *Faci*‡	*#* *Individual*	*#* *Pro*	*#* *Faci*	*#* *Individual*	*Pro*	*Faci*	*Individual*
*1*	*Asian palm civet* *(P. hermaphroditus)*	*40*	*428*	*6598*	*49*	*1474*	*22,105*	*5.2%*	*36.2%*	*35.3%*
*2*	*Malayan porcupine (H. brachyura)*	*44*	*1446*	*23,706*	*45*	*567*	*16,079*	*0.6%*	−*20.9%*	−*9.2%*
*3*	*Hoary bamboo rat (R. pruinosus)*	*25*	*97*	*4672*	*42*	*394*	*29,567*	*13.8%*	*42.0%*	*58.6%*
*4*	*Wild boar (S.scrofa)*	*40*	*453*	*17,852*	*34*	*212*	*6957*	−*4.0%*	−*17.3%*	−*21.0%*
*5*	*Oriental rat snake* *(P. mucosus)*	*39*	*534*	*116,614*	*33*	*393*	*130,004*	−*4.1%*	−*7.4%*	*2.8%*
*6*	*Asian cobra* *(N. naja)*	*40*	*547*	*159,111*	*32*	*540*	*203,607*	−*5.4%*	−*0.3%*	*6.4%*
*7*	*Masked palm civet (P. larvata)*	*21*	*74*	*1117*	*31*	*228*	*5339*	*10.2%*	*32.5%*	*47.9%*
*8*	*Blue peafowl* *(P. cristatus)*	*13*	*44*	*679*	*31*	*114*	*2671*	*24.3%*	*26.9%*	*40.8%*
*9*	*Sambar deer (C.unicolor)*	*32*	*1395*	*5609*	*30*	*1008*	*4596*	−*1.6%*	−*7.8%*	−*4.9%*
*10*	*Siamese crocodile (C. siamensis)*	*34*	*2515*	*934,710*	*28*	*1296*	*936,937*	−*4.7%*	−*15.3%*	*0.1%*
*11*	*Ring-necked Pheasant* *(P. colchicus)*	*34*	*148*	*11,177*	*26*	*63*	*16,222*	−*6.5%*	−*19.2%*	*9.8%*
*12*	*Asian black bear* *(U. thibetanus)*	*30*	*187*	*779*	*25*	*105*	*516*	−*4.5%*	−*13.4%*	−*9.8%*
*13*	*Indian python* *(P. molurus)*	*23*	*1189*	*97,315*	*24*	*283*	*49,369*	*1.1%*	−*30.2%*	−*15.6%*

⁎ CAGR = compound annual growth rate.

† Pro = provinces.

‡ Faci = facilities.

## 4 Discussion

This paper compiled the first analysis of the GoV's national CWFM database. Previously no country-wide statistics had been published on the distribution of CWFs, the number of species, and how many individual animals were being bred and raised, except for the annual sDFP report. All animal classes were identified in CWFs in all provinces, including >450 wildlife species. In 2021, in the Level 1 protection group, there were 95 species being raised at 1824 CWFs; in Level 2, there were 137 species in 4554 CWFs; and in Level 3, there were 139 species in 1499 CWFs. Over the years of the study period, our analysis found an overall decrease in CWFs and numbers of individual animals, but the continued movement, domestication, and exploitation of wildlife species needs to be monitored to assess the risks of disease transmission from zoonoses originating from wildlife.

All provinces had CWFs, but the number of facilities and types of species were not equally distributed. Geographically, this was probably due to the market factors, feed resources, transport, contract farming practices, and local customs. Some species were bred and raised based on their popularity in certain regions. For example, local traditions in Vinh Tương district in Vinh Phuc province supported snake farming (279 CWFs), while deer farming was popular in Quynh Luu district in Nghe An province (179 facilities) and Vinh Cuu district in Dong Nai province (163 facilities). Reptiles were the most numerous species. This could have been due to favorable raising factors such as the successful incubation and hatching in artificial conditions and an abundant food supply. Reptiles such as python, crocodile, and softshell turtle were popular in the SE and MRD regions. Other species were identified in much larger geographic areas, such as porcupine, wild boar, and palm civet. Raising and breeding some species was based on economic development models in some areas, but was market dependent [6,7,9,10].

According to aggregated data of CITES-MA, in 2013 the total number of CWFs was 22,400, with 150 species and approximately 3.3 million individuals. By 2020, the number of CWFs had decreased to 8672, with approximately 2,527,945 individuals. Some of these species in these CWFs were identified as endangered, and included elephants, tigers, and bears. The number of bears decreased from 4300 in 2005 to approximately 317 individuals in 2021 in 105 CWFs [19]. These figures are similar to the figures in our database.

The number of CWFs has fluctuated over time. In addition to market factors, there are also mechanisms and regulations that have changed related to species management. Some species are no longer under the management of the forestry sector [17], or have been transferred to the livestock sector, such as sika deer, wild duck, butterfly lizards, and Chinese water dragons [15]. Other species are now managed by the aquaculture management agency, including softshell turtles and water snakes [12]. Breeding of non-traditional livestock and rare animals, including wild animals, has been encouraged as a household economic development model. Many new animal species have been promoted, imported, domesticated, and introduced into animal husbandry, such as ostriches, Burmese pythons, sika deer, guinea fowl, and crocodiles. Some wildlife species have contributed to the diversification of livestock products. Other species, having a higher value, but facing a niche consumer market, have been negatively affected, compared to traditional livestock species. Breeders of a newly introduced species often face difficulties because the species' health and behavior are not understood, including the prevention and treatment of diseases and the animals' adaptability to captive conditions related to reproduction. Additionally, there have been cases where some farmers and traders have abused the new-species farming model and have over-reproduced and propagated a species until they make a profit, but other farmers have been bankrupted [2,[4], [5], [6], [7]].

The CWFM database is an important, simple, and effective tool that is feasible for use nationwide. It allows the sDFPs to easily implement data collection with accurate and timely information on CWFs to provide up to date information on distribution, type of animals, animal numbers, and protected groups. On a broader scale, the analysis of the data can identify production capacity and support the strategic development of market connections. Relevant stakeholders can be mobilized to share technical information such as animal movement using forest product inventory request and disease surveillance. The forest protection sector can use the data to implement state regulations, including improving the efficiency of registration and licensing management to reduce fraudulent practices by tracing animal and animal products when owners sell animals or animal products using the system's electronic log-book system to record any changes of the animal of the facilities according to the government requirements. The database can be customized in the future to add additional information for wider use such as training, technical guidelines, market information, diseases and biosecurity management, and channels to collect disease and biosecurity management information so as to grant farm licenses. These software features could be promoted when all sDFPs are officially required to use the database, which will allow the local authorities to mobilize their resources for better management of this sector.

There is a need to minimize the public health impact from zoonoses originating from wildlife. Bird (Aves) species and mammal classes have been identified to be more likely to harbor zoonotic viruses than other species, including Corona and Influenza viruses [20,21]. Some common birds that have been sold as pets, such as Starlings, Passerines, Cuckoos, and the Common Hill Myna, were reported in a few provinces during our study. In the MDR there had been previous reports of collection and selling of bat droppings for fertilizer [22,23]. Even though no province reported this in our study, this practice poses a high zoonoses risk as it has been well documented that bats are reservoirs for a number of zoonotic viruses [24].

It is crucial to assess the risks of disease transmission in wildlife breeding, raising and commercial trading by exploring the biosafety and biosecurity practices. Effective risk communication and risk reduction activities need to be activated if threats are identified.

The sDFPs need to strengthen wildlife management activities, including monitoring risky breeding and raising practices. For example, many species of wild animals are kept in the same CWF, and livestock and pets are also kept together. Additionally, in CWFs, contact between animals and humans is very common. The CWFM database can provide information for inter-sectoral risk assessment of disease emergence and spill over by identification of areas or sites with high density of CWFs in close proximity to in residential areas, livestock farms, slaughterhouses, animal markets.

One limitation of this study could have been linked to under or erroneous reporting. Additionally, the fluctuating trends of CWFs we identified could have been due to species not adapting to, or not able to reproduce, in a captive environment.

## 5 Conclusion

This was the first analysis of national data from the CWFM database to provide an up-to-date compilation on the distribution of CWFs, the number of species, and how many individual animals were being bred and raised. There were CWF in all provinces and regions with different distribution, species, and number of animals. The overall number of CWF in 50 provinces decreased by a negative compound annual growth rate of −7.2%, along with a reduction in the number of individual animals bred and raised in these facilities. However, it is crucial to monitor the changing dynamics to assess the risks of disease transmission from zoonoses originating from wildlife to minimize the public health impact. In the future, there is a need to generate more evidence to understand breeding and raising practices to develop guidelines for CWFs. We recommend that the CITES-VNForest should legally require periodic reporting of CWFs via the CWFM database. Information about CWFs needs to be shared publicly so that community members are aware of activities and can participate in monitoring the origin of animals, as well as facilitating market connections for the registered CWFs.

## Funding

This work was conducted with financial support from the 10.13039/100000200United States Agency for International Development (USAID) grant number GHA-G-00-06-00001 through the Emergency Centre for Transboundary Animal Diseases (ECTAD), Food and Agriculture Organization of the United Nations (FAO), Country Office for Vietnam.

## CRediT authorship contribution statement

**Nhu Van Thu:** Conceptualization, Methodology, Investigation, Data curation, Validation, Writing – original draft. **Scott Newman:** Conceptualization, Methodology, Writing – review & editing. **Pawin Padungtod:** Conceptualization, Methodology, Supervision, Writing – review & editing.

## Declaration of Competing Interest

The authors declare the following financial interests/personal relationships which may be considered as potential competing interests:

Food and Agriculture of the United Nation reports financial support was provided by United States Agency for International Development. The authors would like to thank Dr. Vuong Tien Manh and Mr. Nguyen Van Doan MSc., from CITES-MA and all staff of the Sub-Department of Forest Protection who were actively involved in this study by collecting and inputting information into the CWFM database. We thank Dorothy L Southern, the manuscript and publication editor of FAO, for critically reviewing this manuscript.

## Data Availability

No data was used for the research described in the article.
